# Inflammatory Markers Associated with Physical Frailty and Cognitive Impairment

**DOI:** 10.14336/AD.2024.0258

**Published:** 2024-04-17

**Authors:** Yiming Pan, Lina Ma

**Affiliations:** Department of Geriatrics, Xuanwu Hospital Capital Medical University, National Research Center for Geriatric Medicine, Beijing 100053, China

**Keywords:** Frailty, cognitive impairment, inflammaging, chronic inflammation

## Abstract

In older adults, physical frailty and cognitive impairment contribute to adverse outcomes. However, the research on mechanisms underlying physical frailty and cognitive impairment is limited. Low-grade chronic inflammation is a characteristic of aging. Particularly, an imbalance in pro- and anti-inflammatory mechanisms may be involved in frailty and neurodegenerative disorders. Therefore, exploring the inflammatory markers of physical frailty and cognitive impairment is crucial to fully understanding these mechanisms and establishing a substantial link between these two disorders. Notably, few studies have focused on exploring inflammatory markers in both physical frailty and cognitive impairment, posing a major challenge in elucidating the link between them. Therefore, substantial efforts are required for the better prevention of physical frailty and cognitive impairment. In this review, we explored the role of inflammatory markers as a potential link between frailty and cognitive impairment.

## 1. Introduction

The risks of aging-associated decline in physical and cognitive reserves increase with normal aging and the accumulation of chronic diseases. Physical frailty (PF) and cognitive impairment (CI) are two important targets of secondary interventions in aging research for improving independence and life quality in older individuals. Although the relationship between PF and CI remains controversial, epidemiological research has revealed that PF increases the risk of CI and is associated with attention, executive function, and processing speed [1]. Simultaneously, individuals with CI are more likely to become frail [2]. Therefore, it is biologically plausible to consider that PF and CI share a pathophysiological etiology, and the potential mechanisms underlying this link remain under consideration [3]. Chronic inflammation [4], hormone metabolism disorder [3, 5], mitochondrial dysfunction [6], oxidative stress [7], nutrient deficiencies [8], imbalance of the gut microbiota [9], and genetic factors [10] are all considered to be the intersection mechanisms of PF and CI. Exploring common biomarkers between PF and CI may lead to the development of new intervention strategies for maintaining physical and cognitive reserves.

**Figure 1.**
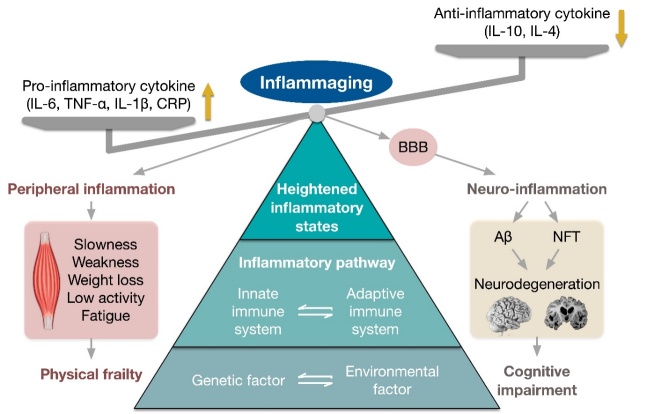
**Hypothetical link between physical frailty, cognitive impairment and inflammaging**. Abbreviations: IL, interleukin; TNF, tumor necrosis factor; CRP, C-reactive protein; BBB, blood-brain barrier; Aβ, Amyloid β; NFT, neurofibrillary tangles.

Aging is linked to immunosenescence, which is a dynamic adaptive process [11] (Fig. 1). Gait speed and physical performance are negatively affected by chronic inflammation [12], even in patients with Alzheimer's disease (AD) and mild cognitive impairment (MCI) [13]. As the central nervous system and immune system constantly interact, an inflammatory response in the cerebrovascular area may trigger a subsequent response in the blood-brain barrier (BBB), releasing inflammatory cytokines into the brain [14]. However, Nogueira et al. found that inflammation was not associated with CI in older adults [15]. Therefore, examining whether inflammation is the link between PF and CI based on literature and further exploring the potential targets to slow the progression to PF and CI in older adults are crucial. This review integrates preclinical and clinical studies related to geriatrics, neurology, and immunology and summarizes the role of various inflammatory cytokines in linking PF to CI.

## 2. Exploring the role of inflammation in PF and CI

### 2.1 Search strategy

The following search terms were used in the PubMed and Web of Science databases from inception through December 2023: inflammation, inflammatory, chronic inflammation, inflammaging, inflammatory index, markers, PF, frailty, frail, CI, cognitive decline, AD, MCI, cognitive frailty (CF), aging, aged, and older adults. Studies written in English were included, regardless of sex, ethnicity, or geographical location. Studies that did not fully report methods or results, as well as conference abstracts, were excluded.

### 2.2 Inflammaging

Inflammation accelerates the aging process by disrupting multiple physiological systems and organism-level function. Further, it strongly influences the pathogenesis and progression of age-related diseases [16-18]. Inflammaging refers to a chronic state of low-grade pro-inflammatory activity as a result of the up-regulation of the inflammatory response driven by multiple age-related factors [19]. The characteristics of inflammaging include increased levels of pro-inflammatory cytokines such as interleukin-1β (IL-1β), IL-6, IL-12, tumor necrosis factor α (TNF-α), as well as C-reactive protein (CRP), and decreased levels of anti-inflammatory cytokines, such as IL-10, IL-4, IL-13, and IL-1 receptor antagonist (IL-1Ra) [20] (Fig. 1). The increased levels of pro-inflammatory cytokines coexist with those of anti-inflammatory cytokines, and the counterbalance between inflammaging and anti-inflammaging determines the “aged-phenotype” [11]. Further, inflammaging can directly or indirectly affect brain function, causing CI [21]. These findings indicate that chronic inflammation is associated with both PF and CI.

### 2.3 Role of inflammation in PF

Inflammation contributes to frailty and pre-frailty [22]. High levels of IL-6, TNF, and CRP are associated with morbidity and mortality [23-26], as well as poor function and mobility status [12, 27-29]. A strong association of serum IL-6, TNF-α, and CRP with lower physical function, muscle strength, and muscle mass has been reported in older adults [30, 31]. Notably, higher IL-6, TNF-α, and CRP levels were observed in older frail adults than those in nonfrail adults of the same age [22, 32]. Exercise intervention can reduce inflammation and improve frailty, mobility performance, and muscle mass in older adults [33, 34] (Fig. 1).

### 2.4 Communication between the central nervous system and immune system: peripheral inflammation and neuroinflammation

Neuroinflammation, a dynamic and complex process in the central nervous system, involves crosstalk between inflammatory components such as cytokines, microglia, and astrocytes [35]. Peripheral inflammatory mediators may increase BBB permeability by changing signaling, enhancing cellular traffic, increasing solute permeability, and directly damaging [36]. Although microglial cells initially migrate toward the BBB to protect its integrity [37], in a state of chronic systemic inflammation, they phagocytose the astrocyte end-feet, triggering the leakage of peripheral inflammatory factors into the central nervous system [37]. Simultaneously, peripheral inflammatory mediators activate the nucleotide-binding oligomerization domain-like receptor family pyrin domain containing 3 (NLRP3) inflammasome, thereby impairing the clearance of Amyloid β (Aβ) by microglia. This promotes the precipitation and hyperphosphorylation of tau proteins, triggering the amyloid cascade and causing irreversible damage to neurons [38-40]. Activated microglia also release pro-inflammatory cytokines, including IL-1β, TNF-α, and IL-6, further aggravating neuroinflammation [41]. In addition, peripheral inflammatory mediators activate astrocytes through dopamine receptor D3 signaling, making them more neurotoxic than neuroprotective, thereby promoting neuroinflammation [42]. In summary, peripheral inflammatory mediators can penetrate the BBB, activate microglia and astrocytes, and trigger neuroinflammation, ultimately leading to neurodegenerative changes [43].

### 2.5 Association between peripheral inflammation and CI

Epidemiological studies on the link between inflammation and cognition have found results that cover the negative to positive spectrum of associations. Individuals with prolonged higher levels of inflammatory proteins from middle age exhibit poorer cognition in older age [44]. Further, individuals with higher levels of inflammatory factors in blood are at an increased risk of CI decades later [45]. High levels of circulating inflammatory proteins such as IL-1β, IL-6, IL-8, IL-13, TNF, neutrophil gelatinase-associated lipocalin, myeloperoxidase (MPO), macrophage inflammatory protein (MIP)-1β, and monocyte chemoattractant protein-1 (MCP-1) may be potential predictive markers for impaired cognition [46-48]. A meta-analysis reported that aerobic exercise can significantly reduce IL-6 and TNF-α levels, and improve CI in patients with MCI or AD. In preclinical models, induced peripheral inflammation activates microglia to produce excess IL-1β and TNF-α, consequently activating astrocytes to produce excess chemokines, with subsequent neuroinflammation and CI [49]. Notably, anti-TNF-α treatment can reduce peripheral inflammation and reverse CI [50]. Chronic and excessive inflammatory responses have been reported in CI associated with AD, vascular dementia, and Lewy body disease. The findings show that inflammation may contribute to CI and that anti-inflammatory interventions can improve cognition and further support the role of inflammation in brain aging.

The pathophysiology of AD involves inflammation [51]. Postmortem histopathological examination of the brains of patients with AD has shown the presence of hyperphosphorylated tau protein, neurofibrillary tangles, and high levels of IL-6 [52]. Patients with CI, even with schizophrenia and major depression, have higher circulating levels of inflammatory markers, including IL-1β, IL-2, IL-4, IL-6, IL-8, IL-10, TNF-α, soluble TNF receptor 1 (sTNFR1), sTNFR2, MCP-1, MCP-3, CRP, and high-sensitivity CRP (hsCRP) [13, 53-55]. However, other studies have not confirmed this relationship [56, 57]. The differences may be related to the progression of CI due to the up-regulation of inflammatory factors, which are likely to appear in the early stages of CI and change in a non-linear manner [56, 57]. Cognitive decline because of peripheral inflammation may involve neuroinflammation caused by the interaction between systemic inflammation and the central nervous system (as discussed in Section 2.3) (Fig. 1); however, the specific underlying mechanism remains unexplored.

## 3. Common inflammatory markers potentially associated with PF and CI

Chronic inflammation is a major cause of age-related diseases and has a negative effect on the aging phenotype and lifespan [16]. An imbalance of pro- and anti-inflammatory mechanisms may lead to poor physical function, further influencing the central nervous system. Therefore, this imbalance may be involved in the mechanisms of frailty and neurodegenerative diseases. This section introduces various inflammatory cytokines that are prominent mediators of both peripheral inflammation and neuroinflammation, potentially linking PF to CI. Notably, their inhibition/activation may be associated with physical and cognitive improvements (Table 1).

**Table 1:** Various inflammatory biomarkers for physical frailty and cognitive impairment.

Inflammation parameter		Physical frailty	Cognitive impairment
Pro-inflammatory cytokine	IL-6	+++	++
	**TNF-a**	+++	++
	**CRP**	+	++
	**IL-1**	+	+
	**Fibrinogen**	+	+
	**sICAM-1**	+	+
Anti-inflammatory cytokine	IL-10	+++	+
	**IL-4**	+	+

Abbreviations: IL, interleukin; TNF, tumor necrosis factor; CRP, C-reactive protein; sCIAM-1, soluble intercellular adhesion molecule-1.

### 3.1 Pro-inflammatory cytokines

#### 3.1.1 IL-6

IL-6 plays a significant role in inflammaging and can serve as a valuable indicator of pro-inflammatory cytokine activation due to its extended duration in comparison to other cytokines. Accordingly, IL-6 has been termed the “cytokine for geriatricians” [58]. IL-6 levels tend to rise with age, independent of other comorbidities [59].

Mice overexpressing IL-6 exhibit muscle atrophy, reduced grip strength, increased frailty index, and an imbalance in muscle mitochondrial homeostasis [60, 61]. Furthermore, high circulating IL-6 levels can predict slow gait speed, poor physical performance, cognitive decline, frailty, disability, and mortality risk in older populations [23, 27, 62-64]. Down-regulation of IL-6 signaling-related genes may reduce the risk of frailty [65]. However, single nucleotide polymorphisms in IL-6 are not associated with frailty or serum IL-6 levels [66]. Therefore, other age-related causes of IL-6 elevation may have a greater impact on physiological frailty than genetic variations. Further, no correlation was found between IL-6 and body composition or function in the Copenhagen Sarcopenia Study [67]. This may be because all participants recruited in the research were characterized as living independently and being apparently healthy, so it cannot be interpreted as IL-6 being unrelated to PF.

Numerous studies have demonstrated a relationship between IL-6 levels and CI development. Higher IL-6 levels are associated with poorer cognitive function and higher risk of death [68]; however, this relationship may be sex-specific [69]. Elevated IL-6 levels can indicate the risk of CI later in life in middle-aged individuals, or in patients with specific diseases such as stroke and diabetes [70-72]. IL-6 is localized in neurons, astrocytes, and microglia in the brain [73-75]. In patients with AD, plasma IL-6 levels are negatively associated with cognitive performance and hypothalamic/hippocampal volume [52]. Chronic inflammation promoted by IL-6 and IL-6R in the central nervous system can lead to neurodegeneration and cause AD [76]. Further, IL-6 may cause microvascular changes, leading to reduced neuronal proliferation, myelin sheath damage, and declined processing speed [77]. Additionally, global brain and hippocampal atrophy [78] as well as muscle atrophy [63] are associated with higher IL-6 levels, which links AD to PF from a pathological perspective. However, other studies found no association between IL-6 levels and longitudinal changes in cognitive function or the risk of CI [79]. Increased soluble IL-6R levels are associated with a reduced dementia risk in the oldest women [80], suggesting that the role of inflammatory factors on cognition may be different in younger-old and the oldest-old individuals. Cognitively healthy older adults with elevated IL-6 levels in the cerebrospinal fluid (CSF) exhibit better cognitive function and a lower AD pathology burden [81], indicating that IL-6 may be protective before the onset of CI. These findings indicate that IL-6 may not be harmful to brain health, depending on the conditions. From a genetic perspective, individuals with the IL-6-174 G/C and IL-6-572 G/C polymorphisms are more likely to develop neuroinflammation that evolves into AD [82]. The IL-6 rs2228145 polymorphism is associated with pathological changes in patients with CI and AD [83]. The IL-6 rs1800795 polymorphism may affect AD susceptibility in Asian populations [84] but not sporadic AD [85].

#### 3.1.2 TNF-α, sTNF1, and sTNF2

TNF-α has been widely implicated in PF and dependency among older adults [64, 67]. TNF-α levels are significantly elevated in people with frailty and sarcopenia [86]. TNF rs1800629 exhibited the strongest association with the frailty phenotype in the English Longitudinal Study of Ageing [87]. In the community population, circulating TNF-α levels are significantly negatively correlated with muscle strength [63]. Exercise intervention could reduce TNF-α levels in myocytes in frail individuals, thereby improving muscle strength [88]. TNF-α is involved in the mammalian target of rapamycin (mTOR) signaling pathway [89]. TNF-α can regulates metabolic processes such as protein synthesis and autophagy [90], and may induce mitochondrial dysfunction [91].

Furthermore, TNF-α expression is up-regulated in the CSF [92], serum [93], and brain [94] of patients with AD. TNF-α levels are negatively correlated with memory performance and gray matter volume in older adults [95]. High circulating TNF-α levels predict decreased muscle strength [63] and cognition [96]. TNF can mediate neuronal necroptosis [97]. Animal experiments have confirmed that anti-TNF-α treatment can reduce peripheral inflammation and microglia activation in the hippocampus [50], improve cognitive function, and reduce the risk of AD [98]. Although TNF-α is considered one of the best inflammatory biomarkers, consensus on its role in PF and CI has not yet been achieved.

Patients with AD or MCI have higher CSF and plasma sTNFR1 and sTNFR2 levels than controls [13]. TNFR1 mainly mediates inflammatory and pro-apoptotic signaling, whereas sTNFR2 has neuroprotective effects and promotes regeneration [99]. sTNFR1 indicates the inflammatory status, distinguishes the degree of CI [100], and predicts memory decline in older adults [69, 101].

#### 3.1.3 CRP and hsCRP

CRP and hsCRP levels are good predictors of physical or cognitive performance and mortality risk in older populations [25, 102, 103]. Peripheral CRP and hsCRP levels are negatively correlated with muscle strength and mass, gait speed, mobility, and independence [27, 62, 63, 104]. Multiple studies have confirmed the correlation between high peripheral CRP or hsCRP levels and PF [104, 105]. Longitudinal studies have shown that higher CRP levels are associated with the risk of frailty in later life [106, 107] and accelerate the progression of frailty [108]. Notably, the CRP levels in frail individuals have been reported to remain high for >10 years [109]. A meta-analysis showed that interventions targeting frailty could reduce CRP levels [110], further confirming the link between CRP and PF. However, this link may be sex-specific and is more pronounced in women [67].

Individuals with higher CRP levels perform worse in cognitive domains such as verbal learning memory, general wakefulness, verbal fluency, and executive function [111, 112], and have smaller hippocampal volumes [113]. Non-demented older adults or patients with lacunar infarction who have higher hsCRP levels present a more severe CI [114, 115]. After adjusting for cognition, a stronger association was observed between high hsCRP levels and PF [116]. In the Honolulu-Asia Aging Study, higher CRP levels were associated with a three-fold increased risk of dementia at 25 years of follow-up [117]. Furthermore, in an 11-year follow-up of the Elderly Asian Community Cohort, after adjusting for common cardiovascular risk factors, serum CRP levels were associated with future vascular dementia [118]. In the Atherosclerosis Risk in Communities cohort study, increased CRP levels in midlife were associated with CI after 20 years [45]. Circulating CRP levels are important markers of cognitive function, whereas the DNA methylation signature of CRP is more strongly associated with brain volume and cognitive function than serum CRP [119, 120]. High levels of circulating CRP and genetically predicted elevated CRP levels can predict the risk of AD [113, 121], which may be caused by CRP mediating neurodegeneration [122].

However, other studies have not confirmed this relationship. In the REasons for Geographic and Racial Differences in Stroke study, elevated CRP levels were used as a marker of CI, but not as a predictor [123]. Some studies have also reported contradictory conclusions. Patients with MCI with low CRP levels are more likely to develop AD dementia [124, 125], while high hsCRP levels have a protective effect on brain structure and cognitive function in patients with CI [125]. Researchers speculate that CRP and hsCRP may show U-shaped changes in the progression of CI. Therefore, older adults with low CRP levels are primarily in the middle of the CI course, while high CRP levels are often observed in the early stages of CI.

#### 3.1.4 IL-1, IL-1α, and IL-1β

Studies on IL-1 and PF are limited. PF is often accompanied by an increase in IL-1β levels [64]; however, this increase may be age-related [126]. Proteomic analysis of frailty has reported IL-1α to be associated with longitudinal worsening of PF [127]. One study found significant differences in the frequency of the A2 allele of the IL-1Ra variable number tandem repeat polymorphism between frail and non-frail groups in an older Mexican population [128].

The serum concentration of IL-1α in patients with AD is significantly reduced [93], whereas IL-1β is significantly increased [129]. IL-1 is associated with many neurotrophic and gliotrophic actions that lead to AD, both *in vitro* and *in vivo* [130]. Chronic inflammation induces microglia to produce IL-1β, which disrupts hippocampal gamma rhythm and inhibits hippocampal plasticity [49, 131], leading to cognitive dysfunction. The neuropathological mechanism of AD may be related to the neurotoxicity of IL-1 [132], which is manifested in that IL-1β may activate tau protein kinases, promote Aβ deposition [133], and induce nitric oxide production in hippocampal cells [134]. IL-1 induces the expression of inducible nitric oxide synthase in astrocytes, which indirectly potentiates N-methyl-D-aspartate-induced neurotoxicity [135]. IL-1α and -β polymorphic forms may be risk factors for AD [136, 137]. IL-1α (rs1800587) and IL-1β (rs1143623 and rs1143634) polymorphisms are significantly associated with AD or cognitive damage [82, 138]. These findings provide initial insights into the potential roles of IL-1 in the pathogenesis of dementia and lay the foundation for further investigation into its function in PF and CI.

#### 3.1.5 Fibrinogen

Elevated fibrinogen levels are a hallmark of frailty and pre-frailty [139]. Frail and pre-frail individuals in the Cardiovascular Health Study exhibited significantly higher fibrinogen levels than controls [140]. Elevated fibrinogen levels are longitudinally associated with frailty, slow gait speed, and low muscle strength [107, 141, 142]. Further, higher peripheral fibrinogen levels are associated with CI risk and severity [143, 144]. However, fibrinogen in the CSF cannot be used as a pathological marker for AD [145]. High baseline fibrinogen levels indicate long-term cognitive deficits [146]. In addition, fibrinogen and Aβ interaction causes increased fibrinogen aggregation, Aβ fibrosis and continued neuroinflammation aggravation by increasing CRP levels ultimately leading to neurodegeneration and increased AD risk [147, 148]. However, fibrinogen can prevent Aβ from interacting with cells and thereby reduce its adverse effects [149].

#### 3.1.6 Soluble intercellular adhesion molecule 1 (sICAM-1)

sICAM-1 plays a role in leukocyte emigration and activates pro-inflammatory cascades by enhancing IL-6, TNF-α, and MIP-2 production *in vitro* [150, 151]. A gradual increase in sICAM-1 has been observed in non-frail, pre-frail, and frail individuals. A multivariate multinomial logistic analysis revealed a significant association between sICAM-1 levels and frailty; however, the association was independent of IL-6 levels [152]. Higher sICAM-1 levels have been reported in patients with AD and vascular dementia [100, 153]. In mice, changes in hippocampal neurogenesis and Y-maze performance were significantly negatively correlated with sICAM-1 serum levels [154]. In individuals with normal cognition, high sICAM-1 levels in CSF are associated with reduced perfusion levels in the parietal cortex and AD pathology, thereby mediating CI [155, 156]. Increased sICAM-1 secretion from astrocytes reduced Aβ load, blocked NF-κB up-regulation, provided neuroprotection, and improved cognitive performance in AD animal models [157].

### 3.2 Anti-inflammatory cytokines

#### 3.2.1 IL-10

IL-10 is an important anti-inflammatory cytokine. Both circulating IL-10 and IL-10 mRNA in muscles increase with age [126, 158]. The active suppression of the immune response with aging may be beneficial to a certain extent [159]. Populations carrying minor alleles of the IL-10 rs1800871 and rs1800896 polymorphisms are more likely to develop PF [160]. Similar to humans, aged mice exhibited a systemic increase in IL-10 levels [159]. IL-10 knockout (IL-10 KO) mice characterized by increased weakness and decreased muscle strength with age are considered a model of frailty [161]. IL-10 deficiency increased the expression of nuclear factor-κB-induced inflammatory mediators [162] and reduced muscle energy metabolism [163]. Furthermore, weakness and accelerated muscle loss in IL-10 KO mice were ameliorated by anti-inflammatory resveratrol-rich grape seed extract [164].

CSF IL-10 levels exhibit a U-shaped relationship with age, with an inflection point (lowest level) at 50 years [165]. IL-10 levels in the CSF of patients with AD are higher than controls [13]. Elevated IL-10 levels in older adults are associated with poor executive function, slow processing speed, and MCI [79, 166]. Although IL-10 levels cannot predict future MCI risk [79], they can predict the occurrence of long-term CI in ICU survivors [167]. Notably, Rolipram improved CI by reducing TNF-α and increasing IL-10 levels in the hippocampus in diabetic rats [168]. Further, depression-like behavior, cognitive deficits, and enhanced neuroinflammation in IL-10 KO mice improved upon pretreatment with IL-10 [169]. Moreover, IL-10 disrupted Aβ proteostasis, leading to impaired memory in Amyloid Precursor protein transgenic mice, suggesting a complex interplay between innate immunity and proteostasis [170]. Therefore, investigating whether IL-10 expression or mRNA is reduced in the serum or skeletal muscle is crucial to supporting its role in muscle loss and cognitive decline in older adults [171].

#### 3.2.2 IL-4

Few studies have investigated the role of IL-4 in frailty and CI. The combined IL-4^low^-IL-1Ra^high^ genotype is a marker of frailty syndrome risk [128]. IL-4 levels are higher in the CSF of patients with AD than in those without dementia, and AD progression is inversely correlated with IL-4 levels [172]. IL-4 has potential neuroprotective and repair effects on the brain, particularly in the hippocampal region [173, 174]. IL-4 promotes the expression of CD36 and Aβ-degrading enzymes to induce Aβ clearance by microglia [175], drives high Arg1 expression in microglia, and triggers brain-derived neurotrophic factor-dependent neurogenesis [176]. IL-4 gene polymorphisms may influence the risk of AD [177]. Further investigations into the role of IL-4 in aging are warranted, particularly in patients with poor physical activity or cognitive function.

## 4. Multivariate biomarkers as an alternative option

Peripheral inflammation and neuroinflammation are complex processes because various circulating inflammatory cytokines may interact with each other. Given the complexity of the inflammatory network, as mentioned in Section 3, it is difficult to determine the best biomarker linking PF to CI. Therefore, use of a combination of biomarkers may enhance the strength and consistency of these associations. However, attempts to develop a comprehensive set of inflammatory markers to maximize the ability to predict functional outcomes remain limited [178-181]. Notably, multivariate biomarkers may be more beneficial for predicting PF and CI than a single inflammatory mediator.

### 4.1 Inflammatory index

The inflammatory index score (IIS), an additive index of serum IL-6 and sTNFR1 levels, can effectively indicate age-related chronic inflammation and predict 10-year mortality in older adults [178]. Frail and pre-frail individuals exhibited a higher IIS than non-frail controls [182]. IIS can also predict the risk of mortality in patients with end-stage renal disease and older HIV-infected or uninfected injection drug users [183, 184]; however, a larger population is required for analysis. Although the predictive ability of IIS with respect to PF and CI is still unknown, prospective application of IIS is particularly appealing because serum IL-6 and sTNFR1 levels can be obtained easily and inexpensively, and the index is simple and reliable.

### 4.2 Combined inflammatory markers

An index integrating seven circulating inflammatory factors (CRP, IL-1β, IL-1Ra, IL-6, IL-18, TNF-β1, and TNF-α) developed by Bandeen-Roche et al. was independently associated with mobility limitation and frailty risk [179]. These findings indicated that systemic inflammation can be validly measured using a biologically informed summary of inflammatory markers [179]. Principal component analysis (PCA) has been applied in the Health, Aging, and Body Composition study to identify optimal combinations of inflammatory markers associated with physical function, resulting in the identification of two principal components from eight inflammatory factors: one related to TNF-α and the other to CRP [185]. These biomarkers were positively associated with walk time and negatively with grip strength [185]. In the InCHIANTI study, PCA has been performed to reveal biological relationships among inflammatory markers and their significant roles in chronic diseases [180].

Furthermore, partial least squares-discriminant analysis (PLS-DA) was used to classify patterns of inflammatory markers in different physical performances [181]. Calvani et al., using six discriminant biomarkers, found that higher levels of IL-8, MPO, and TNF-α were associated with slower gait speed in older adults. Further, PLS-DA can reduce the dimensionality of data and has reliable and accurate prediction performance, so it is more suitable for exploring longitudinal changes in inflammatory markers with frailty [186]. Marzetti et al. reported that increased CRP and decreased MPO, IL-8, MCP-1, and platelet-derived growth factor-BB levels are the core inflammatory features of physical frailty and sarcopenia (PF&S) [187]. Picca et al. found that patients with PF&S exhibited increased sICAM-1, tissue inhibitor of metalloproteinases-1, and TNF-α levels and decreased glial fibrillary acidic protein and IL-6 levels [188]. Mitchell et al., using longitudinal mixed models to analyze non-targeted proteomic data from older women, identified eight core pro-inflammatory proteins related to frailty and its longitudinal progression [189]. The application of multidimensional data processing provides a new method for exploring the combination of PF and CI inflammatory markers.

### 4.3 Biomarker-based frailty index (FI-B)

The FI-B includes 40 biomarkers (inflammatory, hematological, immunological, cell senescence, genetic, and epigenetic). FI-B can predict mortality more effectively and robustly than any individual biomarker. Notably, FI-B performed better than the clinical deficit FI in predicting mortality risk. Further, FI-B may also help elucidate potential biological interactions between frailty and aging [190]. However, measuring 40 biomarkers is complex and expensive, limiting the wider applications of FI-B.

## 5. Future suggestions

Taken together, although numerous epidemiological and experimental studies have provided evidence linking frailty to cognitive decline, research directly exploring the mechanisms underlying this link is insufficient.

### 5.1 Definition and inflammatory markers of CF

CF refers to the existing parallels between aging, PF, and CI [191]. Results from the Three-City Study, Italian Longitudinal Ageing Study, and Thought Study suggest an association between frailty and incident non-AD dementia, indicating that PF probably represents a physiological state occurring before non-AD CI [192-194]. However, Kelaiditi et al. defined CF as a condition independent of dementia or preexisting brain disorders, although both conditions share several pathophysiological mechanisms and risk factors [191]. Moreover, an association between CF and AD has also been reported [195]; however, these diseases have different neurophysiologies [196]. Therefore, the definition of CF remains debatable. Furthermore, many questions, including the chronological order of the development of PF and CI and any epidemiological evidence for the progression of CF toward dementia, still need to be answered before identifying biomarkers of CF. Additionally, whether higher levels of peripheral inflammatory markers indicate CF remains unknown. Notably, emerging biomarkers of PF and CI may help establish a more reliable definition of CF.

### 5.2 Cutoff point for inflammatory biomarkers of frailty and pre-frailty

Although numerous biomarkers of frailty have been identified, to date, no biological marker has been proposed for clinical diagnosis. Moreover, no consensus has been achieved regarding the cutoff points for the levels of inflammatory markers for prediction, and the available data, as discussed in Section 3, are conflicting. Some markers have been significantly associated with frailty but not pre-frailty, implying that the increase in inflammation is more pronounced from pre-frailty to frailty than from robust to pre-frailty [116]. Hence, identifying biomarkers of pre-frailty is important for the early prediction of frailty.

### 5.3 Different frailty measurements and genetic background

The association between inflammatory markers and frailty or cognition is inconsistent across studies owing to the measurement of different biomarkers and cognitive functions. Walker et al. demonstrated a greater likelihood of CI in individuals with repeatedly high levels of inflammation for >20 years [45], indicating that repeated measurements of blood-based biomarkers and multiple cognitive functions are required for consistent inferences [197]. Although the lack of consensus on a frailty assessment tool is still a major problem, the findings were generally consistent across the two major models (Fried phenotype and Frailty Index) in several studies. The effect of the balance between pro- and anti-inflammatory factors may also be influenced by the genetic background. For example, carriers of the APOEε4 allele exhibited decreased CRP levels [113], whereas higher CRP levels in non-carriers of the APOEε4 allele were associated with better cognition [122]. Further, significant differences in IL-6 and interferon γ levels were found between low- and high-risk MCI groups and controls stratified by APOEε4 status [198], illustrating the importance of genetic factors.

### 5.4 Potential intervention strategies targeting inflammation for PF and CI

An unhealthy lifestyle seriously threatens the health of older adults. Extended sitting can cause low-grade chronic inflammation [199], lead to muscle loss[200], affect cognitive function[201], and increase mortality from inflammatory diseases[202]. Physical exercise can effectively reduce systemic inflammation [203] and have positive impacts on PF [204] and CI [205, 206] in older adults. Pro-inflammatory diets are associated with the occurrence of PF [207] and CI [208]; therefore, low-inflammatory diets can be used as preventive or interventional measures for PF and CI. In addition, studies have shown that dietary and circulating n-3 polyunsaturated fatty acids (PUFA) have anti-inflammatory effects [209, 210]. Increasing n-3 PUFA in the diet may reduce the risk of PF [211, 212] and have protective effects on cognition [213].

Intervention measures such as probiotic intake [214], oxygen-ozone therapy [215], and mesenchymal stem cell transplantation [216] are under preliminary research and are expected to treat CF through anti-inflammatory mechanisms. The effects of anti-inflammatory drugs on CF are still uncertain [217-219].

## 6. Conclusion

In this review, we support the hypothesis that inflammatory markers are the potential links between PF and CI. PF and CI may share common biomarkers; however, the markers associated with PF and CI have not been consistent across different studies. Two reasons for this discrepancy are that many studies are not comparable because of the different measurement methods of frailty, cognition, and inflammation parameters, and the populations in different studies are not homogeneous. Longitudinal studies or clinical trials with large samples across sex, age, race, and different populations may help to explain the common pathways of inflammation in PF and CI. The application of multiple clinical interventions targeting inflammation is also one of the new strategies for treating CF. Although many questions remain unanswered regarding this issue, our review provides a basis for further studies to validate different inflammatory markers for predicting CF risk in older individuals.
